# Study on the Correlation Between Aggressive Behavior and Gut Microbiota and Serum Serotonin (5-HT) in Working Dogs

**DOI:** 10.3390/vetsci12060526

**Published:** 2025-05-28

**Authors:** Ning Sun, Liuwei Xie, Jingjing Chao, Fuxiao Xiu, He Zhai, Yuanting Zhou, Xi Yu, Yingyi Shui

**Affiliations:** 1 School of Police Dog Technology, Criminal Investigation Police University of China, Shenyang 110048, China; xieliuwei@cipuc.edu.cn (L.X.); cindy304@163.com (J.C.); daxiu881123@126.com (F.X.); zhaihe1984@126.com (H.Z.); 18621504719@163.com (Y.Z.); 2 Police Dog Team, Criminal Investigation Corps, Shanghai Public Security Bureau, Shanghai 201799, China; 3 Tongling Public Security Bureau, Tongling 404100, China; squallyuxi@126.com; 4 School of Information and Cyber Security, People’s Public Security University of China, Beijing 100038, China; xxsyy0531@163.com

**Keywords:** canine, aggressive behavior, gut microbiota, 5-HT, offensive aggression, defensive aggression

## Abstract

Aggressive canine behavior poses a significant threat to public health. Understanding aggressive behavior is crucial for canine socialization and human–dog interactions. This study conducted an exploratory analysis of working dogs to investigate changes in gut microbiota and neurotransmitters associated with aggressive behavior. Notably, it represents the first research to systematically differentiate canine aggression into offensive and defensive subtypes for investigation. In this study, 56 working dogs from three regions of China, comprising different breeds (11 Spaniels, 13 German Shepherds, and 32 Belgian Malinois), aged 4.89 ± 1.54 years, and of both sexes (38 males and 18 females), were assessed and grouped for aggressive behavior using a C-BARQ-based questionnaire. Then, 16S rRNA sequencing and ELISA were employed to compare differences in gut microbiota and serotonin concentrations between aggressive (n = 35) and non-aggressive (n = 21) groups, as well as between offensive (n = 26) and defensive (n = 9) aggression subgroups. β-diversity analysis confirmed no significant correlation between aggressive behavior and gut microbiota composition (*p* > 0.05), suggesting a limited role of gut microbiota in modulating host behavior. Comparative analysis of gut microbiota composition revealed no significant differences in phylum-level abundance among different aggression types (*p* > 0.05). Notably, the non-aggressive group exhibited significantly higher relative abundances of *Escherichia-Shigella*, *Erysipelotrichaceae_UCG-003*, and *Clostridium_sensu_stricto_1* compared to the aggressive group (*p* < 0.05). Random forest analysis identified *Lactobacillus* as a biomarker for canine aggressive behavior and *Turicibacter* as a discriminatory factor between offensive and defensive aggression. The results demonstrated a strong correlation between aggression and 5-HT neurotransmission. Serum serotonin levels were significantly lower in both the defensive (39.92 ± 2.58 ng/mL) and offensive (50.07 ± 3.90 ng/mL) aggression groups compared to the non-aggressive group (59.49 ± 2.76 ng/mL), with the lowest levels found in defensively aggressive dogs. The defensive group showed significantly lower serotonin levels than the offensive group (*p* < 0.001). The results demonstrate that different behavioral phenotypes in aggressive dogs lead to distinct gut microbiome compositions. This suggests that microbiome analysis may facilitate early diagnosis and preventive intervention before aggressive behavior manifests. As such, 5-HT shows potential as a monitoring tool for diagnosing canine aggression, with significant practical applications in canine behavior management.

## Simple Summary

In recent years, the frequent occurrence of dog attacks has resulted in thousands of children suffering bite injuries annually, making canine aggressive behavior a topic of significant concern in human society. Understanding this behavior is crucial for canine socialization and human–dog interactions. This study conducted an exploratory analysis of working dog populations to investigate alterations in gut microbiota and neurotransmitters associated with aggressive behavior with the aim of clarifying causal relationships influencing canine aggression and identifying potential approaches for its management and mitigation. The results demonstrate that distinct behavioral phenotypes in aggressive dogs lead to variations in gut microbiome composition, suggesting that microbial profiles may facilitate diagnostic and preventive interventions prior to the manifestation of aggressive behaviors. Notably, serotonin (5-HT) emerges as a potential biomarker for monitoring and diagnosing canine aggression, offering valuable applications for public safety management.

## 1. Introduction

The domestic dog (*Canis familiaris*) has coexisted with humans for at least 15,000 years [1]. As one of the most popular companion animals, dogs have developed a close relationship with humans [2]. Dogs stand as the sole domesticated large carnivorous species, with fear responses, aggressive tendencies, and tameness having been historically regarded as pivotal traits in their domestication process [3]. As a natural component of canine behavioral repertoire, aggressive behavior serves critical adaptive functions in territorial defense, social dominance establishment, predation, mate competition, and reproductive interactions [4]. However, in human society, canine aggression has become a significant concern. Aggressive behaviors directed at humans account for 54–67% of all aggression cases [5], representing the most common and dangerous behavioral problem. Numerous dog bite incidents occur annually [6], causing physical injuries, psychological trauma, disease transmission, and even fatalities to both humans and other dogs [7,8]. Canine aggression has become a serious threat to public health in some countries. From an animal welfare perspective, aggression is a leading cause of euthanasia or abandonment [9].

The serotonergic neurotransmitter system is key in regulating aggression across species [10]. Notably, 5-hydroxytryptamine (5-HT, serotonin), a crucial neurotransmitter for mood and emotion regulation [11], shows dysregulation in the central nervous system of dogs with low aggression thresholds [11,12]. Low 5-HT levels increase dopamine and norepinephrine, lowering aggression thresholds and enhancing impulsivity [13]. Aggressive dogs consistently demonstrate reduced 5-HT levels [11,14]. Notably, over 90% of 5-HT is produced by specialized enterochromaffin cells, mucosal mast cells, and enteric neurons in the gut [15]. These gut cells exhibit neuron-like properties and modulate central nervous system function through the enteric nervous system (ENS) and vagus nerve [16]. This bidirectional gut–microbiota–brain interaction constitutes the gut–brain axis [17], which consists of neural pathways (ENS, vagus, sympathetic, and spinal nerves) and humoral pathways (cytokines, hormones, and neuropeptides) [18]. Growing evidence indicates gut microbiota influences host behavior via the gut–brain axis [19], particularly in exploration, neophobia, sociability, stress, and anxiety-related behaviors [19,20]. Studies across species—mice [21], hamsters [22], laying hens [23], and dogs [24,25]—confirm microbiota–aggression associations. Kirchoff et al. [26] collected fecal samples from 31 bulldogs and performed next-generation sequencing of the bacterial 16S rRNA V3-V4 region. Differences in the β diversity of the gut microbiota between aggressive and non-aggressive dogs demonstrated that canine aggression is related to the gut microbiota composition. Previous research primarily attributed aggressive behavior to low 5-HT levels and elevated cortisol concentrations [27], as cortisol is considered a biomarker for anxiety- and aggression-related behavior [28]. However, Mondo et al. [25] investigated the gut microbiome structure and adrenal cortex activity in dogs with aggressive and phobic behavioral disorders. Their findings indicated that the gut microbiota and metabolites showed stronger correlations with aggressive behavior compared to the effects of hormones such as testosterone and cortisol. Furthermore, aggressive dogs exhibited distinct gut microbial profiles compared to both phobic and normal behavior groups. Craddock et al. [24] also confirmed differences in gut microbiota composition between aggressive and non-aggressive dogs. Additionally, Pellowe et al. [29] reported increased microbial richness in aggressive and anxious dogs. In summary, these studies reveal significant correlations among neurotransmitter activity, gut microbiota composition, and aggressive canine behavior.

Working dogs play vital roles in national and international crime/terrorism prevention. For centuries, humans have capitalized on dogs’ exceptional olfactory sensitivity for odor detection, with canines demonstrating detection thresholds below parts-per-trillion (1 × 10⁻^12^) levels [30]. Dogs’ extraordinary olfactory capabilities have been effectively utilized in multiple specialized fields, including narcotics detection [31], explosives identification [32], human remains localization [33], search and rescue operations [34], and medical diagnostics (particularly cancer detection) [35]. Additionally, working dogs—particularly large, powerful breeds with innate aggression and biting capabilities—are extensively deployed in patrols, crowd control, hostage rescue missions, and the apprehension of terrorists. Unlike companion dogs, working dogs require controlled aggression [36] and fearlessness [37]—fear being the primary disqualifier in selection [38]. Common working breeds (German Shepherds, Belgian Malinois, Rottweilers, Dobermans, and Chinese Kunming Dogs) exhibit inherent aggression. Existing research has demonstrated associations between canine behavioral disorders (e.g., aggression and anxiety) and specific gut microbiota profiles [25,26]; however, research on the relationship between gut microbiota, serotonin (5-HT), and aggressive behavior in working dogs remains remarkably scarce. Understanding canine aggression is crucial for socialization and human interaction, yet challenging due to its biological complexity. This exploratory study examines gut microbiota and neurotransmitter changes associated with aggression in working dogs, aiming to clarify causal relationships and develop management strategies.

## 2. Materials and Methods

### 2.1. Animals

A total of 56 working dogs participated in this study, including 38 males and 18 females. The breeds consisted of 11 Springer Spaniels, 13 German Shepherds, and 32 Belgian Malinois, with an average age of 4.89 ± 1.54 years. Each dog was housed individually in a separate kennel with free access to water. As aggression is influenced by multiple factors, we collected various parameters for each dog, including breed, age, sex, and dietary information. These parameters served as covariates in our analysis (Table 1). All dogs were healthy and in good condition, with no gastrointestinal symptoms or antibiotic treatments within the two months before fecal sample collection. The working dogs were sourced from police K-9 units in three Chinese regions: 28 from Shenyang (fed Pedigree Innovet dog food), 24 from Shanghai (fed Purina Pro Plan dog food), and 4 from Anhui (fed Pets Guard dog food). The canine diet composition is detailed in Table 2.

### 2.2. Assessment of Aggressive Behavior

Questionnaires were distributed to the owners of all 56 working dogs to collect data on aggressive behaviors and classify dogs based on behavioral phenotypes. The questionnaire was adapted from the C-BARQ scale [39], which shows the strong positive correlation between its aggression/fear components and canine behavioral diagnoses [40]. Our modified questionnaire incorporated working dog-specific characteristics with two key improvements: An expanded 30-item scale with refined 0–10 scoring (0 = none, …, 10 = severe) for more precise assessment of responses to standard stimuli and the inclusion of fear-related behavioral indicators in a 0–10 scale to differentiate between offensive and defensive aggression. Statistical analysis using SPSS 22.0 (n = 56) showed a Cronbach’s α = 0.961 (>0.8 threshold), indicating high internal reliability. KMO = 0.839 (0.8–0.9 range), confirming significant inter-variable correlations. These results validate the questionnaire’s internal consistency reliability and structural validity for discriminating aggressive behaviors. The complete questionnaire is detailed in Table 3.

### 2.3. Sample Collection

For the fecal collection, from May to September 2024, 56 fresh fecal samples were collected by aseptic technique within 1 h after defecation and immediately stored in a refrigerator at −80 °C.

For blood collection, blood samples (5 mL) were collected from the cephalic vein of each dog, put into tubes without anticoagulant, centrifuged at 37 °C for 10 min at 3000× *g* to analyze the serum samples, and finally stored in a refrigerator at −20 °C.

### 2.4. Extraction of Microbial Genomic DNA

We extracted fecal microbial DNA using the E.Z.N.A™ Mag-Bind Soil DNA Kit (Omega Bio-tek, Norcross, GA, USA), following the manufacturer’s instructions. Subsequently, we assessed DNA integrity via agarose gel electrophoresis (AGE).

### 2.5. PCR Amplification

The bacterial 16S rRNA gene in fecal samples was amplified via PCR using universal primers (314F and 805R). The primers were fused with sequencing platform-compatible 16S V3-V4 adapters with the following sequences: 314F: 5′-CCTACGGGNGGCWGCAG-3′; 805R: 5′-GACTACHVGGGTATCTAATCC-3′. The PCR reaction mixture consisted of 15 μL of 2× Hieff^®^ Robust PCR Master Mix, 1 μL each of forward and reverse primers, 10 ng of template DNA, and ddH₂O to a total volume of 30 μL. PCR amplification was performed under the following conditions: Initial denaturation at 94 °C for 3 min; 5 cycles of denaturation at 94 °C for 30 s, annealing at 45 °C for 20 s, and extension at 65 °C for 30 s; 20 cycles of denaturation at 94 °C for 20 s, annealing at 55 °C for 20 s, and extension at 72 °C for 30 s; and final extension at 72 °C for 5 min, followed by holding at 10 °C. Index adapters were ligated to the ends of the 16S rRNA amplicons for NGS sequencing. Each sample underwent two rounds of PCR amplification.

### 2.6. Library Quality Control, Pooling, and High-Throughput Sequencing

The DNA libraries were assessed for quality and quantified. Library size and concentration were determined using 2% AGE and the Qubit^®^ 4.0 Fluorometer, respectively. All samples were pooled in equimolar ratios (1:1). The libraries were denatured and sequenced on the Illumina MiSeq platform.

### 2.7. 5-HT Measurement

The 5-HT levels were measured using a 5-HT-ELISA kit (CEA808Ge 96T, Wuhan, China). Intra-assay coefficient of variation (CV): <10%. Inter-assay CV: <12%. Detection range: 11.11–900 ng/mL.

### 2.8. Data and Statistical Analysis

After data filtering and quality control, sequences were clustered into operational taxonomic units (OTUs) at 97% similarity using Usearch 11.0.667. The taxonomic composition and abundance of microbial communities in each sample were analyzed at different classification levels. The rarefaction curve of *α*-diversity indices was generated using R v3.6.0 to assess species richness and evenness. The *α*-diversity indices were calculated using mothur v1.43.0. The PCA plot was generated using R v3.6.0, while the PCoA plot was created with the vegan package in R, both calculating the contribution rates of their respective principal components.

Using OriginPro 8.5, the standard curve equation was plotted based on a four-parameter log-logistic model, incorporating the standard concentrations and OD values from the ELISA kit. The four-parameter log-logistic model was employed for curve fitting, expressed by the equation y = A2 + (A1 − A2)/[1 + (x/x0)^*p*], where A2 represents the curve’s lower asymptote (the limit as x approaches positive infinity), A1 denotes the upper asymptote (the limit as x approaches negative infinity), x0 indicates the inflection point (the x-value at half-maximal response), and *p* governs the curve’s slope and shape. The 5-HT concentrations in samples were calculated accordingly. All data are expressed as mean ± sd deviation. In all statistical analyses, *p* < 0.05 was considered statistically significant.

## 3. Results

### 3.1. Sample Sequencing Data

Questionnaire results classified 21 dogs as non-aggressive and 35 as aggressive, with 26 exhibiting offensive aggression and 9 exhibiting defensive aggression. In total, 56 canine fecal samples were collected and sequenced on the Illumina MiSeq platform. After denoising, 5,711,382 high-quality sequences were obtained, with an average of 100,199.68 ± 42,978.67 sequences per sample. Specifically, the aggressive group contained 100,363.72 ± 42,354.43 sequences, while the non-aggressive group had 99,918.48 ± 45,084.50 sequences. After quality control, the sequence characteristics were as follows: Mean length (MeanLen): 415 bp, minimum length (MinLen): 353 bp, and maximum length (MaxLen): 416 bp. The aggressive group showed an average length of 414.13 ± 4.92 bp, while the non-aggressive group averaged 415.79 ± 6.50 bp.

### 3.2. α-Diversity Analysis

**Figure 1:**
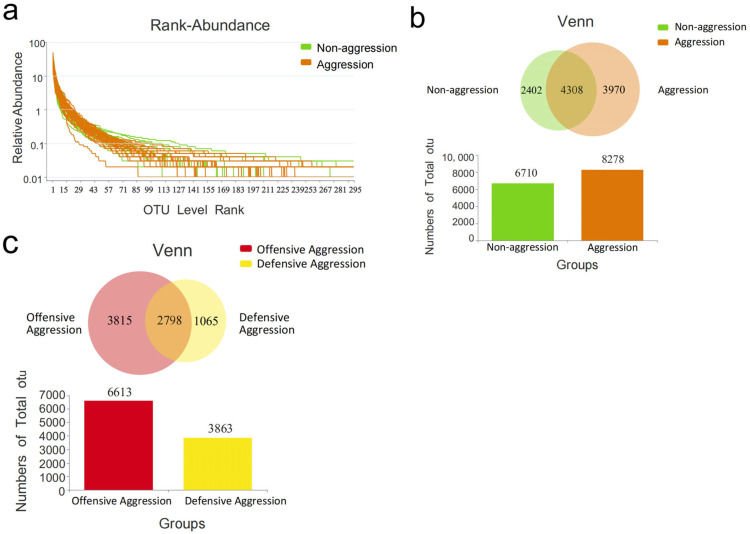
α- diversity analysis diagram. (**a**) Rank–abundance curve (*x*-axis: OTU rank by relative abundance; *y*-axis: abundance value). (**b**,**c**) Venn diagrams comparing OTU distributions.

The obtained rank–abundance curves (Figure 1a) reflect species richness and evenness. The aggressive group exhibited a longer tail on the *x*-axis, indicating higher bacterial richness than the non-aggressive group. As seen in the Venn diagram in Figure 1b,c, the aggressive group had 8278 total OTUs (3970 unique). The non-aggressive group had fewer OTUs than the aggressive group. The offensive aggression group contained 6613 total OTUs (3815 unique), exceeding the defensive aggression group (Figure 1c).

Table 4 shows that there were no significant differences (*p* > 0.05, *t*-test) in α-diversity indices (Ace, Chao, Shannon, and Simpson) between the aggressive vs. non-aggressive or offensive vs. defensive subgroups. Coverage indices exceeded 99% for all samples, confirming sufficient sequencing depth and reliable microbial community detection.

### 3.3. β-Diversity Analysis

**Figure 2:**
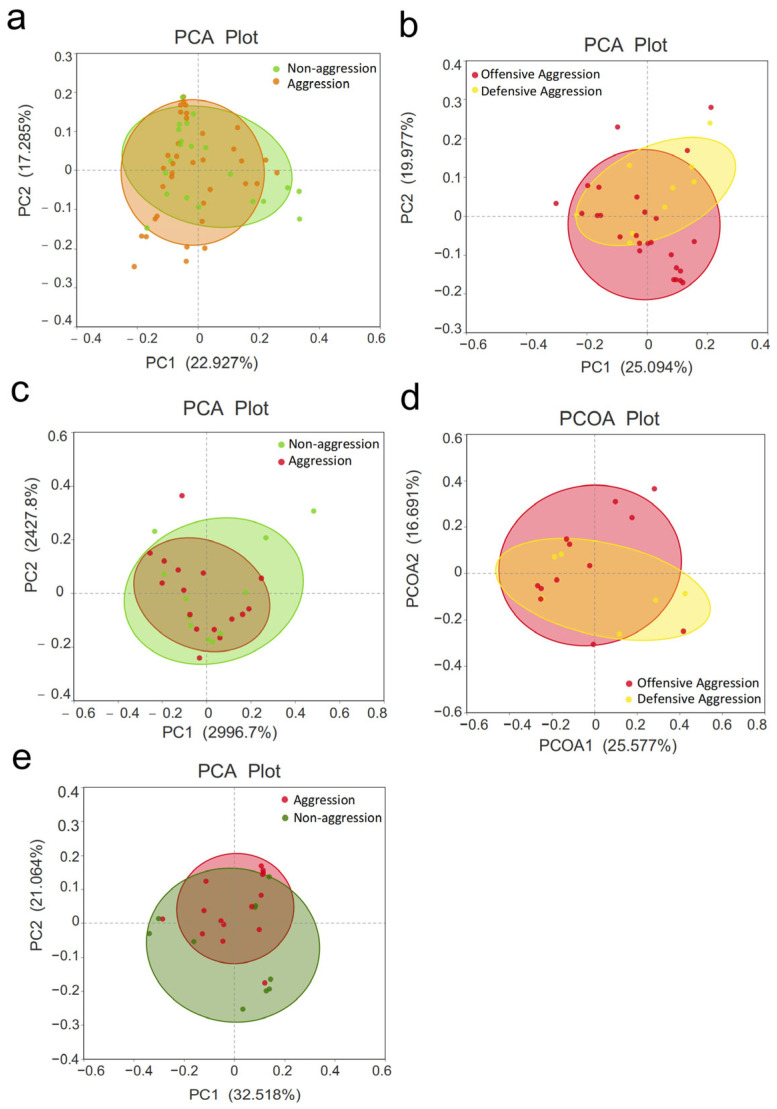
PCA plots of β-diversity across aggression-related groups. (**a**) Aggression/Non-aggression (all dogs); (**b**) Offensive Aggression/Defensive Aggression (all dogs); (**c**) Aggression/Non-aggression (Shenyang); (**d**) Offensive Aggression/Defensive Aggression (Shenyang); (**e**) Aggression/Non-aggression (Shanghai). Note: The axes represent selected principal components with percentages indicating their explanatory power for compositional variance; the scales are relative distances without practical significance. Same as below.

*β*-Diversity was assessed using Principal Component Analysis (PCA) and Principal Coordinate Analysis (PCOA) to compare microbial community differences among aggression-related groups. The PCA ellipses showed no clear separation between the aggressive and non-aggressive groups (Figure 2a), indicating no significant difference in microbial composition (*p* = 0.755, *R* = −0.026). Slight separation was observed but remained statistically insignificant between the offensive and defensive subgroups (*p* = 0.972, *R* = −0.143) (Figure 2b). Comparisons were also made between dogs from the same geographic region. In the Shenyang samples, no significant differences were found between aggressive and non-aggressive dogs (*p* = 0.102, *R* = 0.082) or between the offensive and defensive subgroups (*p* = 0.175, *R* = 0.100) (Figure 2c,d). In Shanghai samples, weak separation occurred between the aggressive and non-aggressive groups but the difference was not significant (*p* = 0.365, *R* = 0.007) (Figure 2e). Due to the small sample in the defensive group, comparisons between the offensive and defensive groups were not performed.

**Figure 3:**
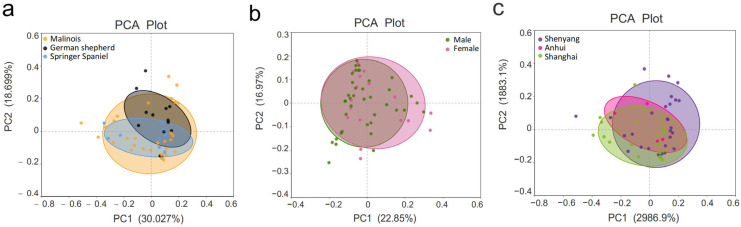
PCA plot of β-diversity among covariate groups.(**a**) Intergroup comparisons by variety; (**b**) Intergroup comparisons by gender; (**c**) Intergroup comparisons by region.

Additional covariates were analyzed for their association with fecal microbial composition. No significant correlations were found between gut microbiota composition and dog breed (*p* = 0.912, *R* = −0.069), sex (*p* = 0.228, *R* = 0.031), or geographical location (*p* = 0.658, *R* = −0.019). See Figure 3.

### 3.4. Composition of Gut Microbiota

#### 3.4.1. Comparison Between Aggressive and Non-Aggressive Groups

**Figure 4:**
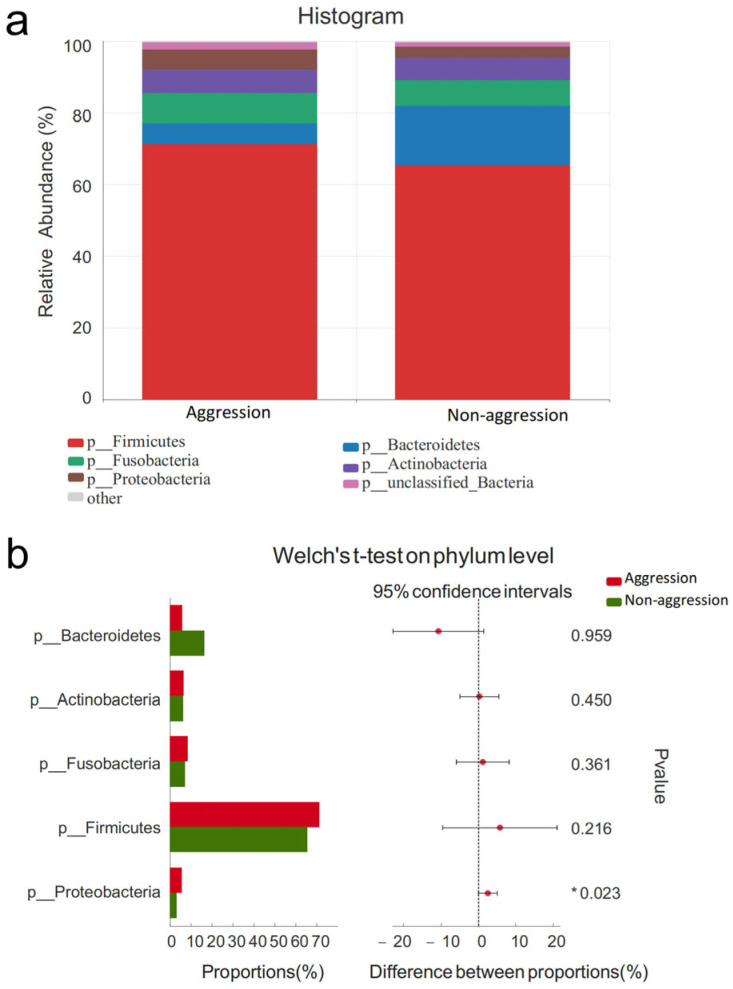
Comparison of gut microbiota characteristics at phylum levels between aggressive and non-aggressive dogs. Note: (**a**) shows species composition at phylum levels; (**b**) displays Welch’s *t*-test results at phylum levels. * indicates significant differences (*p* < 0.05).

At the phylum level, Firmicutes, Fusobacteria, Bacteroidetes, Actinobacteriota, and Proteobacteria were the dominant phyla (relative abundance >1%) in all 56 fecal samples (Figure 4a). Welch’s *t*-test analysis revealed differences in the relative abundance of these dominant phyla between aggressive and non-aggressive dogs. No significant differences were observed in the relative abundances of Firmicutes, Fusobacteriota, Bacteroidota, or Actinobacteriota between the two groups (*p* > 0.05). However, the aggressive group exhibited a significantly higher relative abundance of Proteobacteria (*p* = 0.023, *p* < 0.05), as shown in Figure 4b.

**Figure 5:**
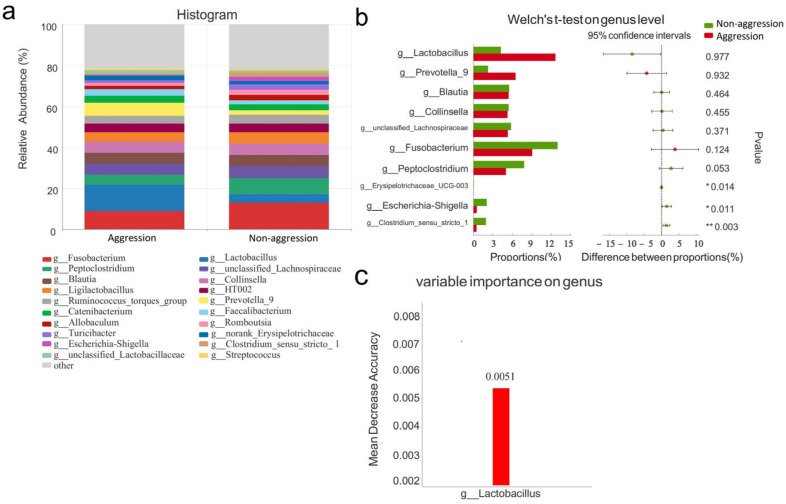
Comparison of gut microbiota characteristics at genus levels between aggressive and non-aggressive dogs. Note: (**a**) shows species composition at genus levels; (**b**) displays Welch’s *t*-test results at genus levels; (**c**) presents the species importance ranking plot, with the *y*-axis indicating importance metrics and ranked species names and the *x*-axis showing importance measurement values/standard deviations. Same as below. * indicates significant differences (*p* < 0.05); ** indicates highly significant differences (*p* < 0.01).

At the genus level, the dominant genera (relative abundance > 5%) in the aggressive group included *Fusobacterium*, *Lactobacillus*, *Peptoclostridium*, *Prevotella_9*, *unclassified_Lachnospiraceae, Blautia*, and *Collinsella* (Figure 5a). When comparing the aggressive and non-aggressive groups, no significant differences were observed in the relative abundances of these dominant genera (*p* > 0.05, Figure 5b). Significant differences were observed in less abundant general, with the top 20 species including *Escherichia-Shigella*, *Clostridium_sensu_stricto_1*, and *Erysipelotrichaceae_UCG-003* (*p* < 0.05) (Figure 5b).

To identify biomarker taxa for canine aggressive behavior, we applied random forest machine learning to genus-level data. This analysis evaluates sample attributes across multiple decision trees to efficiently identify the most discriminative taxonomic features. The random forest model identified *Lactobacillus* as the primary discriminant factor for aggressive behavior. Mean Decrease in Accuracy (MDA) = 0.0051 (Figure 5c).

**Figure 6:**
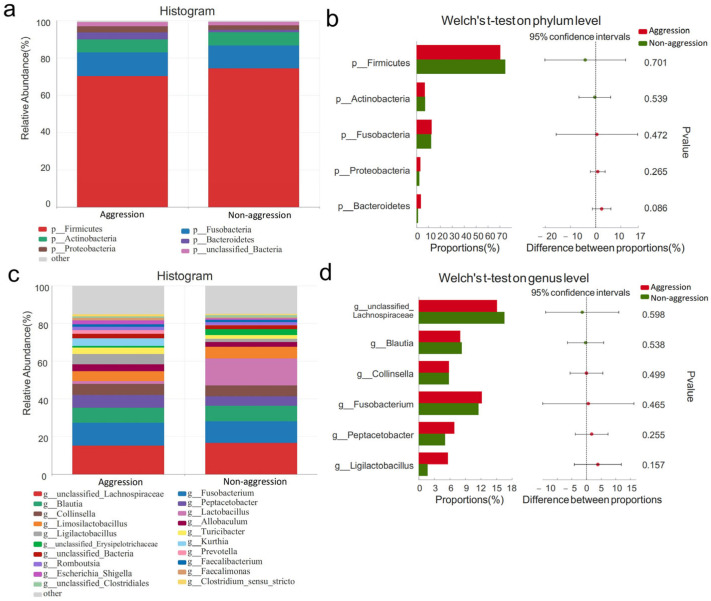
Comparison of gut microbiota characteristics at phylum and genus levels between aggressive and non-aggressive dogs in Shenyang. Note: (**a**,**c**) show species composition at phylum and genus levels, respectively; (**b**,**d**) display Welch’s *t*-test results at phylum and genus levels, respectively.

At the phylum level (Figure 6a), a comparative analysis of gut microbiota between the aggressive (n = 12) and non-aggressive (n = 8) Malinois dogs (n = 20) in Shenyang was performed using Welch’s *t*-test. No significant differences were observed in the relative abundances of dominant phyla between the two groups (*p* > 0.05, Figure 6b). At the genus level, the aggressive group showed the following dominant genera (relative abundance > 5%): *unclassified_Lachnospiraceae*, *Fusobacterium*, *Blautia*, *Peptacetobacter*, *Collinsella*, and *Ligilactobacillus* (Figure 6c). Comparative analysis revealed no statistically significant differences in these dominant genera between the aggressive and non-aggressive groups (*p* > 0.05, Figure 6d).

#### 3.4.2. Comparison Between Offensive and Defensive Aggression Groups

**Figure 7:**
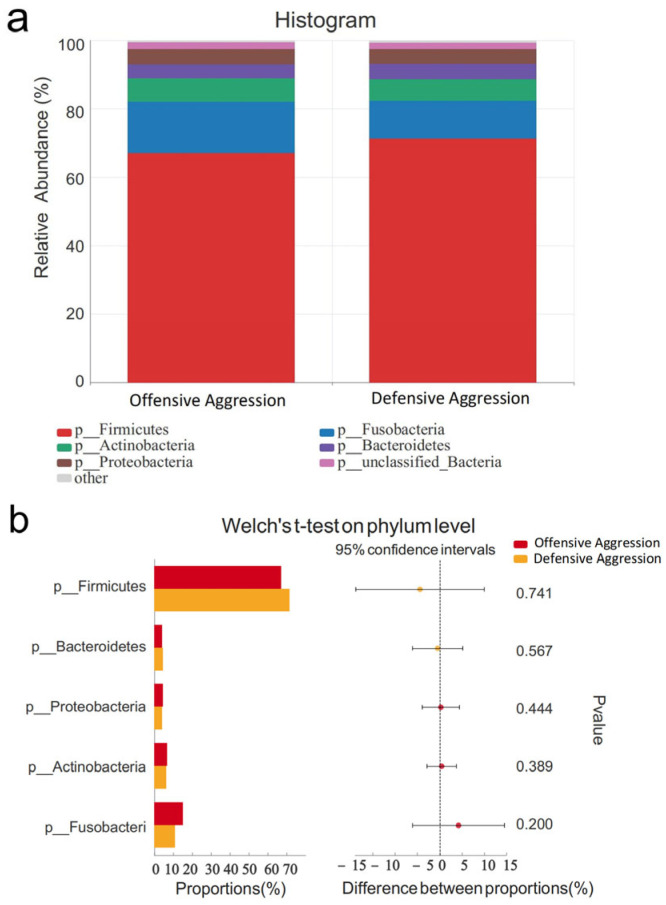
Comparison of gut microbiota characteristics at phylum levels between offensive and defensive aggression. Note: (**a**) shows species composition at phylum levels; (**b**) displays Welch’s *t*-test results at phylum levels.

At the phylum level, the dominant microbiota (relative abundance > 1%) were Firmicutes, Fusobacteria, Actinobacteriota, Bacteroidetes, and Proteobacteria (Figure 7a). No significant differences were observed between offensive and defensive aggression groups (*p* > 0.05, Figure 7b).

**Figure 8:**
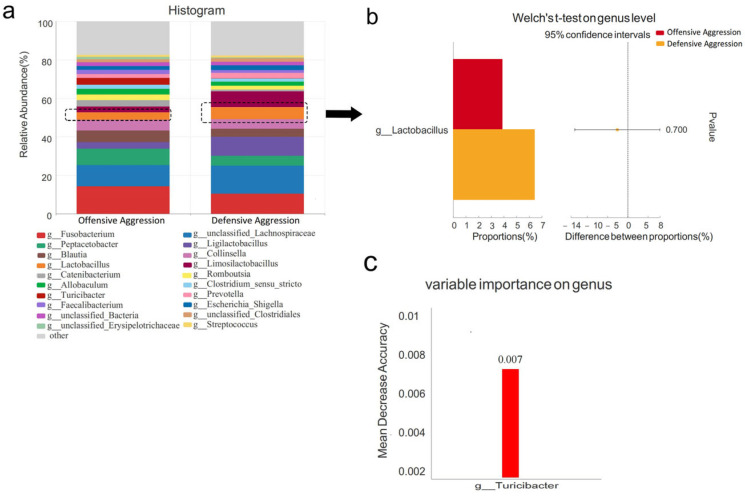
Comparison of gut microbiota characteristics at genus levels between offensive and defensive aggression. Note: (**a**) shows species composition at genus levels; (**b**) displays Welch’s *t*-test results at genus levels; (**c**) presents the species importance ranking plot.

At the genus level, the dominant microbiota in offensive and defensive aggression groups were *Fusobacterium*, *unclassified_Lachnospiraceae*, *Peptacetobacter*, *Ligilactobacillus*, *Blautia*, *Collinsella*, and *Lactobacillus*, respectively (Figure 8a). Although *Lactobacillus* served as a distinctive marker between aggressive and non-aggressive groups, no significant difference was found between offensive and defensive aggression (*p* > 0.05), with higher levels of defensive aggression (Figure 8b). Random Forest analysis identified *Turicibacter* as the key discriminator between the offensive and defensive groups (MDA = 0.007). Welch’s *t*-test confirmed that *Turicibacter* was significantly higher in the offensive aggression group (*p* < 0.01, Figure 8c).

### 3.5. 5-HT Levels

**Figure 9:**
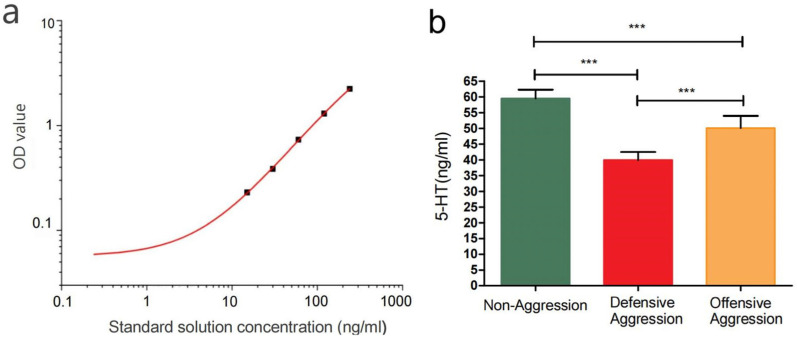
Determination of serum 5-HT concentration. Note: (**a**) shows 5-HT nonlinear standard curve; (**b**) displays comparison of serum 5-HT concentrations among non-aggressive, offensive aggression, and defensive aggression groups. *** indicates extremely significant differences (*p* < 0.001).

The standard curve was generated by measuring the OD values of serial dilutions (S0–S5: 0, 15, 30, 60, 120, and 240 ng/mL) from the ELISA kit, followed by nonlinear regression analysis using Origin 2019 software to establish the calibration curve (Figure 9a). The fitted equation was calculated as y = 7.92 − 7.86/[1 + (x/607.63)^1.03], with a correlation coefficient of *R*^2^ = 0.9992. By substituting the OD values of serum samples into this standard curve equation, the concentrations of 5-HT in the serum were ultimately determined.

The measured concentrations were as follows: Non-aggressive group (59.49 ± 2.76 ng/mL), defensive aggression group (39.92 ± 2.58 ng/mL), and offensive aggression group (50.07 ± 3.90 ng/mL). Both aggression subgroups showed significantly lower 5-HT levels than the non-aggressive group (*p* < 0.001), with the lowest concentrations observed in defensive aggression groups (Figure 9b).

## 4. Discussion

This study focused on working dogs, comparing the gut microbiomes of 35 aggressive and 21 non-aggressive dogs from three geographical regions. β-diversity analysis confirmed no significant correlation between canine aggressive behavior and gut microbiota composition. Considering that the canine gut microbiota is influenced by breed genetics [41], habitat [42], and dietary [43] factors, as well as gender-specific effects on both microbiota composition and aggressive behavior [44], we selected dogs from the same region, maintained identical housing conditions, and provided standardized diets to minimize nutritional and environmental variations. Comparative analyses were conducted separately for 28 dogs from Shenyang and 24 dogs from Shanghai. Despite efforts to homogenize source differences and account for covariates (gender, breed, and region), β-diversity analysis still showed no significant differences in aggression-related gut microbiota. While Mondo et al. [25] reported that increased gut microbial richness and diversity were associated with enhanced canine aggression, our data from rank–abundance curves, Venn diagrams, and α-diversity indices revealed no significant differences between aggressive and non-aggressive groups. The absence of distinct gut microbial profiles across different behavioral phenotypes suggests the existence of a stable “core” gut microbiome, indicating that variations in aggressive behavior are not caused by microbial dysbiosis. Although the gut microbiota’s role in modulating host behavior appears limited, microbial communities can still influence behavioral regulation. This phenomenon is commonly observed in social animals (both primates and non-primates), where significant individual behavioral differences exist within populations, despite generally more homogeneous gut microbiomes at the group level [45,46].

The experimental results demonstrated no significant changes in the relative abundance of bacterial phyla among different aggression types. In the fecal samples from all 56 experimental dogs, the five most abundant bacterial phyla were Firmicutes, Fusobacteria, Bacteroidetes, Actinobacteriota, and Proteobacteria. This finding aligns with previous reports which consistently identified these five phyla as dominant, regardless of variations in canine age, diet, genetic background, living environment, or health status [24,25,26]. Earlier studies presented divergent findings: Kirchoff et al. [26] reported higher Firmicutes abundance in aggressive dogs and elevated Proteobacteria and Fusobacteria levels in non-aggressive counterparts, while Craddock et al. [24] characterized working dog microbiomes and identified increased Firmicutes in aggressive individuals. However, our data revealed no statistically significant differences in Firmicutes, Proteobacteria, or Fusobacteria abundance between aggressive and non-aggressive groups. Previous research has documented notable variations in Bacteroidota relative abundance among dogs displaying behavioral issues including aggression, separation anxiety, and phobias [25,47]. Bacteroides species are known to metabolize tryptophan into imidazole derivatives and short-chain fatty acids (SCFAs) [48]. Dysbiosis-induced reductions in Bacteroides may consequently decrease tryptophan and its derivative serotonin. Although our aggression group showed a lower relative abundance of Bacteroidota, this difference did not reach statistical significance.

When comparing the 56 experimental dogs in this study, we specifically analyzed subsets of dogs with matched geographical origin, living environment, diet, breed, and similar age to control for potential confounding factors affecting gut microbiota. A focused analysis of 20 Malinois dogs from Shenyang revealed that their gut microbial composition—including dominant phyla and genera—was consistent with the larger cohort of 56 dogs. Importantly, at both the phylum and genus taxonomic levels, no statistically significant differences in relative abundance were detected between aggressive and non-aggressive groups in either the Shenyang subset or the full study population.

This study revealed that microbial signatures associated with aggressive behavior are not solely determined by abundance variations, but rather may depend on functional specificities of particular taxa. For instance, while *Eryapiedrichaceae_UCG-003* showed only minor proportional differences, it demonstrated exceptionally high statistical significance (*p* = 0.014), suggesting its potential role in modulating host neural activity through tryptophan metabolism (e.g., the kynurenine pathway) [49,50]. Conversely, *Fusobacterium* exhibited more substantial proportional variations that failed to reach significance (*p* = 0.124), likely reflecting its high inter-individual variability—consistent with previous reports linking this genus strongly to dietary protein intake [51]. Notably, *Lactobacillus* also displayed significant variability, potentially attributable to strain-specific effects on host behavior: certain strains produce inhibitory neurotransmitters like γ-aminobutyric acid (GABA) that modulate aggression [52], while others promote inflammation through lactate production [53], resulting in marked fluctuations at the genus level. Furthermore, evidence indicates resource competition between *Lactobacillus* and other genera (e.g., *Escherichia* and *Bacteroides*) [54,55], with its abundance being dynamically regulated by community structure. Although conventional significance testing detected no notable differences in *Lactobacillus* abundance between the aggressive and non-aggressive groups (*p* = 0.977), random forest modeling identified it as a distinctive biomarker differentiating canine aggression. This discrepancy stems from the model’s capacity to capture variable interactions and nonlinear relationships, whereas univariate analyses focus exclusively on taxon abundance. In microbiome research, machine learning approaches outperform univariate methods in detecting functionally relevant microbial consortia, suggesting that Lactobacillus effects may be mediated through inter-taxa interactions. Consequently, screening for microbial markers of aggressive behavior should integrate both statistical significance and functional specificity. Future investigations should incorporate larger cohorts combined with metagenomic functional profiling and germ-free animal models to validate causal relationships.

Recent studies have associated *Lactobacillus* with various neurological disorders, including anxiety/depression, autism spectrum disorder, multiple sclerosis, and Alzheimer’s disease [56]. Mondo et al. [25] observed elevated *Lactobacillus* levels in phobic dogs, while Kirchoff et al. [26] reported a higher abundance of 25 OTUs within this genus in aggressive dogs. Pellowe et al. [29] similarly documented increased *Lactobacillus* richness in both aggressive and anxious dogs, aligning with findings from Mondo et al. [25] and Craddock et al. [24]. Notably, Kirchoff et al. [26] attributed aggression to higher *Lactobacillus* abundance, whereas Mondo et al. [25] linked its enrichment to fear-related behavior. This discrepancy may stem from classification methodologies for canine aggression, which typically categorizes such behaviors into the following categories: Dominance-related aggression, fear-related aggression, food/possession-related aggression, and territorial aggression [57]. We further stratified aggressive groups into offensive aggression (dominance-related) and defensive aggression (fear-related) subtypes for refined microbial analysis.

The neurobiological mechanisms underlying canine aggression remain incompletely understood. Physiologically, aggression manifests in two primary forms: defensive aggression, which is emotionally driven and difficult to voluntarily control, and offensive aggression, which is purposeful and devoid of emotional signaling [58]. Both forms involve strong autonomic nervous system activation [59]. This study represents the first microbial species-level comparison between offensive and defensive aggression subtypes in dogs. Offensive aggression aligns with dominance-related behavior, while defensive aggression correlates with fear-related responses. In working dog training, the behavioral manifestations of offensive and defensive aggression are distinct and should be differentiated. Defensive aggression typically shows preliminary signs before aggression. For example, when humans approach, the dog may display submissive postures (such as crouching, ears pulled back, tail tucked between legs, or submissive urination). If the perceived threat persists and the dog cannot retreat or escape, it may exhibit aggressive reactions [60]. Offensive aggression often lacks these warning signs, with such dogs launching aggression without apparent precursors. Defensive dogs usually aggress in a restrained and tense manner, using just enough bite intensity and duration to escape the frightening situation. Moreover, compared to offensive aggression, defensive aggression dogs are generally easier to control. Fear is the root cause of this aggressive behavior—eliminate the fear and the aggressive behavior will cease. This highlights how handlers should interpret canine behavior to prevent biting incidents. Some animal behavior experts emphasize that most aggressive behaviors displayed by privately owned dogs are associated with fear/anxiety [12]. For working dogs, fear is an undesirable trait as it reduces work efficiency and safety. This represents a significant issue requiring resolution, necessitating measures to prevent and mitigate this undesirable characteristic in working dogs.

The comparative analysis revealed no significant differences at the phylum level between offensive and defensive aggression groups. However, at the genus level, random forest analysis identified Turicibacter as the primary discriminant factor differentiating between offensive and defensive aggression. Turicibacter is a well-documented producer of short-chain fatty acids (SCFAs) [61]. These SCFAs exert influence on the central nervous system through their interaction with G protein-coupled receptors (GPRs), specifically free fatty acid receptors (FFARs), located on enteroendocrine cells [62].

Low 5-HT levels and imbalanced 5-HT receptor expression are key factors contributing to aggressive behavior. Compared to other neurotransmitter systems, the serotonergic system demonstrates the most consistent activity about the neurobiological mechanisms of aggression [63]. Studies in humans and various animal species have established a clear relationship between 5-HT levels and aggressive behavior [12]. In our study, both the defensive aggression group (39.92 ± 2.58 ng/mL) and offensive aggression group (50.07 ± 3.90 ng/mL) showed significantly lower 5-HT levels than the non-aggressive group (59.49 ± 2.76 ng/mL), indicating distinct serotonergic system differences between aggressive and non-aggressive dogs. These findings align with previous research measuring 5-HT in cerebrospinal fluid [12], urine [64], serum [14], plasma, and platelets [65] of aggressive dogs. Regardless of sample type, aggressive dogs consistently exhibited lower 5-HT concentrations.

Notably, dogs displaying defensive aggression showed the lowest 5-HT levels in our experiment. The defensive group’s 5-HT levels were significantly lower than the offensive group’s, corroborating findings by Rosado et al. [65] and Leon et al. [11], who similarly observed the lowest 5-HT values in defensively aggressive dogs. One study found that offensive dogs, particularly those aggressing without warning signals, had lower concentrations of 5-hydroxyindoleacetic acid (5-HIAA), the primary 5-HT metabolite, in their cerebrospinal fluid compared to non-aggressive dogs [12]. Research suggests that 5-HT-mediated behavioral modifications likely played a crucial role in early domestication by transforming fear-based defensive reactions into offensive responses toward humans [66]. The serotonin 1A and 2A receptor genes (htr1A and htr2A) in the canine serotonergic system have been identified, with htr1A appearing particularly important in dog domestication [67]. Animal models consistently demonstrate the crucial role of the brain’s serotonin system in responding to stressors and generating fear and anxiety [68]. Numerous studies across species have shown that increased 5-HT can reduce aggression while enhancing submissive behaviors [69]. Consequently, serotonergic drugs are employed in treating phobias in both humans [70] and dogs [71].

## 5. Conclusions

This study compared the gut microbiota structure and serotonin (5-HT) levels in working dogs based on their exhibited aggression levels. While no significant correlation was found between aggressive behavior and gut microbiota composition, suggesting a limited role of intestinal microbiota in modulating host behavior, the research holds essential practical implications for canine applications. The results indicate that different behavioral phenotypes in aggressive dogs lead to distinct gut microbiome profiles, implying that the microbiome could aid in diagnosing and preventively intervening against aggression before its manifestation. The gut microbiota may contribute to varying types of aggressive behavior, and modulating it (e.g., through probiotic administration) could potentially mitigate aggression. Additionally, aggression showed a strong association with the neurotransmitter 5-HT, which may serve as a potential tool for identifying and monitoring aggressive tendencies in dogs. Future studies should involve larger cohorts incorporating diverse dog breeds to elucidate the underlying mechanisms linking gut microbiota, neurotransmitters, and aggressive behavior.

## Figures and Tables

**Table 1:** Information of 56 dogs.

Sample-Id	Sex (Male/Female)	Age (Years)	Birth	Breed	Dog Aggression (√/×)	Offensive Aggression/Defensive Aggression (OA/DA)	Region	Diet
A1	M	4.4	1 November 2020	Malinois	√	DA	Shenyang	Pedigree Innovet
A2	M	4.4	1 November 2020	Malinois	√	DA		
A3	M	4.4	1 November 2020	Malinois	√	OA		
A4	M	4.2	20 December 2020	German shepherd	√	OA		
A5	M	4.2	20 December 2020	German shepherd	√	OA		
A6	M	3.8	13 June 2021	Malinois	√	OA		
A7	M	3.8	13 June 2021	Malinois	√	OA		
A8	M	3.8	13 June 2021	Malinois	√	OA		
A9	F	3.6	23 August 2021	German shepherd	×	\		
A10	F	3.6	23 August 2021	German shepherd	√	DA		
A11	F	3.1	16 February 2022	Malinois	×	\		
A12	M	4.0	27 March 2021	Malinois	√	OA		
A13	M	3.8	9 June 2021	Malinois	√	OA		
A14	M	4.2	14 January 2021	Malinois	×	\		
A15	F	4.4	1 November 2020	Malinois	√	DA		
A16	M	4.4	1 November 2020	Malinois	√	DA		
A17	M	4.4	1 November 2020	Malinois	√	OA		
A18	M	4.4	1 November 2020	Malinois	√	OA		
A19	M	4.2	20 December 2020	German shepherd	√	DA		
A20	M	4.2	20 December 2020	German shepherd	√	OA		
A21	F	3.5	27 September 2021	German shepherd	√	OA		
A22	F	3.8	9 June 2021	Malinois	×	\		
A23	F	3.8	9 June 2021	Malinois	×	\		
A24	M	4.2	14 January 2021	Malinois	×	\		
A25	F	3.5	27 September 2021	German shepherd	×	\		
A26	M	4.2	14 January 2021	Malinois	×	\		
A27	F	4.2	14 January 2021	Malinois	×	\		
A28	F	3.1	16 February 2022	Malinois	×	\		
B4	M	5.5	1 October 2019	Malinois	×	\	Anhui	Pets Guard
B1	M	3.9	1 May 2021	Malinois	√	DA		
B2	M	3.9	1 May 2021	Malinois	√	OA		
B3	M	6.6	1 September 2018	Malinois	√	OA		
C1	M	9.4	11 November 2015	Malinois	√	OA	Shanghai	Pro Plan
C2	M	4.9	10 May 2020	German shepherd	√	OA		
C3	F	12.8	11 June 2012	German shepherd	√	OA		
C4	M	9.1	17 February 2016	Springer Spaniel	√	OA		
C5	F	2.2	7 January 2023	Malinois	√	OA		
C6	M	5.0	24 March 2020	Malinois	√	DA		
C7	M	3.9	20 April 2021	Malinois	√	DA		
C8	M	5.4	27 October 2019	German shepherd	√	OA		
C9	M	7.6	1 September 2017	Malinois	√	OA		
C10	M	9.6	22 August 2015	Springer Spaniel	√	OA		
C11	M	9.6	22 August 2015	Springer Spaniel	√	OA		
C13	F	4.8	5 June 2020	German shepherd	√	OA		
C14	F	6.3	13 December 2018	Malinois	√	OA		
C15	M	6.3	2 December 2018	German shepherd	√	OA		
C16	M	7.1	28 January 2018	Springer Spaniel	×	\		
C17	F	2.2	7 January 2023	Malinois	×	\		
C18	M	7.1	28 January 2018	Springer Spaniel	×	\		
C19	F	2.2	7 January 2023	Malinois	×	\		
C20	M	5.5	18 September 2019	Springer Spaniel	×	\		
C21	F	6.3	12 December 2018	Springer Spaniel	×	\		
C22	F	2.7	27 June 2022	Springer Spaniel	×	\		
C23	M	2.7	27 June 2022	Springer Spaniel	×	\		
C24	M	2.7	27 June 2022	Springer Spaniel	×	\		
C25	M	7.1	28 January 2018	Springer Spaniel	×	\		

Note: “√ “ indicates aggressive behavior in dogs, while ”×” denotes non-aggressive behavior.

**Table 2:** Nutrient composition of dog food.

	Content (%)	Crude Protein	Crude Fat	Crude Fiber	Crude Ash	Calcium	Total Phosphorus	Lysine
Dog Food								
Pro Plan		≥31%	≥20%	≤3%	≤10%	≥1%	≥0.8%	≥1.2%
Pedigree Innovet		≥24%	≥14.5%	≤5%	≤10%	\	≥0.6%	\
Pets Guard		≥32%	≥18%	≤4%	≤10%	≥1.2%	≥1.0%	≥1.2%

**Table 3:** Canine Aggressive Behavior Scale (7-Dimension Version).

A. Aggression Toward Familiar People A1. Does your dog exhibit aggressive behavior when verbally corrected or punished (e.g., scolded, yelled at) by you or your colleagues? A2. Does your dog exhibit aggressive behavior when being bathed or groomed by your colleagues? A3. Does your dog exhibit aggressive behavior when stared at directly by a colleague? A4. Does your dog exhibit aggressive behavior when a colleague steps over it?
B. Aggression Toward Strangers B1. Does your dog exhibit aggressive behavior when an unfamiliar adult approaches it directly while it is being walked or exercised on a leash? B2. Does your dog exhibit aggressive behavior when an unfamiliar child approaches it directly while it is being walked or exercised on a leash? B3. Does your dog exhibit aggressive behavior when strangers approach you or your colleagues outside the team? B4. Does your dog exhibit aggressive behavior when strangers approach its kennel? B5. Does your dog exhibit aggressive behavior when strangers attempt to touch or pet it? B6. Does your dog exhibit aggressive behavior when encountering joggers, cyclists, rollerbladers, or skateboarders during a walk?
C. Aggression Toward Familiar Dogs C1. Does your dog exhibit aggressive behavior when another familiar dog is in its kennel? C2. Does your dog exhibit aggressive behavior when another familiar dog approaches its favorite resting/sleeping spot? C3. Does your dog exhibit aggressive behavior when approached by another familiar dog while eating? C4. Does your dog exhibit aggressive behavior when another familiar dog approaches it while it is playing with or chewing its favorite toy, bone, or object?
D. Aggression Toward Unfamiliar Dogs D1. Does your dog exhibit aggressive behavior when an unfamiliar male dog approaches it while it is being walked or trained on a leash? D2. Does your dog exhibit aggressive behavior when an unfamiliar female dog approaches it while it is being walked or trained on a leash? D3. Does your dog exhibit aggressive behavior when an unfamiliar dog enters its territory? D4. Does your dog exhibit aggressive behavior when barked at, growled at, or lunged at by other unfamiliar dogs?
E. Food or Object-Related Aggression E1. Does your dog exhibit aggressive behavior when a colleague takes away its toy, bone, or other objects? E2. Does your dog exhibit aggressive behavior when approached by a colleague while eating? E3. Does your dog exhibit aggressive behavior when a colleague removes its food? E4. Does your dog exhibit aggressive behavior when you or a colleague retrieves food or objects it has stolen?
F. Territory-Related Aggression F1. Does your dog exhibit aggressive behavior toward strangers approaching it while it is in a car (e.g., at a gas station)? F2. Does your dog exhibit aggressive behavior when a stranger approaches you or your colleagues within the team? F3. Does your dog exhibit aggressive behavior when strangers walk past its kennel? F4. Does your dog exhibit aggressive behavior when cats, birds, or other animals enter its kennel? F5. Does your dog exhibit aggressive behavior when unfamiliar people are in its kennel?
G. Pain or Fear-Related Aggression G1. Does your dog exhibit aggressive behavior when injured and sent for treatment? G2. Does your dog exhibit aggressive behavior when it feels afraid? G3. Does your dog exhibit aggressive behavior when forced to do something?

**Table 4:** Alpha diversity index.

Sample		No. of OTUs	No. of Unique OTUs	Ace	Chao	Shannon	Simpson	Coverage%
Non-aggression		6710 ^A^	2402 ^A^	1326.17 ^A^	1232.45 ^A^	3.41 ^A^	0.11 ^A^	99%
Aggression		8278 ^A^	3970 ^A^	1363.63 ^A^	1294.92 ^A^	3.52 ^A^	0.09 ^A^	99%
	Offensive Aggression	6613 ^A^	3815 ^A^	1195.71 ^A^	1110.75 ^A^	3.49 ^A^	0.10 ^A^	99%
	Defensive Aggression	3863 ^A^	1065 ^A^	1264.02 ^A^	1181.87 ^A^	3.56 ^A^	0.08 ^A^	99%

Note: Superscript letters in column A indicate non-significant differences (*p* > 0.05).

## Data Availability

All the data presented in the study are included in the article; further inquiries can be directed to the corresponding authors.
